# Tibolone increases bone mineral density but also relapse in breast cancer survivors: LIBERATE trial bone substudy

**DOI:** 10.1186/bcr3097

**Published:** 2012-01-17

**Authors:** Nigel J Bundred, Peter Kenemans, Cheng Har Yip, Matthias W Beckmann, Jean-Michel Foidart, Piero Sismondi, Bo von Schoultz, Rena Vassilopoulou-Sellin, Rachid El Galta, Eugenie Van Lieshout, Mirjam Mol-Arts, Juan Planellas, Ernst Kubista

**Affiliations:** 1 Department of Surgery, University of Manchester, Southmoor Road, Manchester, M23 9LT, UK; 2 Department of Obstetrics and Gynaecology, VU University Medical Center, PO Box 7057, 1007 MB Amsterdam, The Netherlands; 3 Department of Surgery, University Malaya Medical Centre, Pusat Perubatan Universiti Malaya, Lembah Pantai, 59100, Kuala Lumpur, Malaysia; 4 Department of Surgery, Universitätsklinikum Erlangen, Postfach 2306, D-91012 Erlangen, Germany; 5 Department of Obstetrics and Gynecology, University of Liege, 81 bd de la Constitution, B-4020 Liege, Belgium; 6 Department of Gynecological Oncology, University of Turin, C.So Svizzera, 185 - 10149 Torino, Italy; 7 Department of Obstetrics and Gynecology, Karolinska Institutet, SE-171 77, Stockholm, Sweden; 8 Department of Pathology, The University of Texas MD Anderson Cancer Center,1515 Holcombe Blvd., Houston, TX 77030, USA; 9 Research Data and Quantitative Sciences, Schering-Plough, P.O. Box 20, 5340 BH, Oss, The Netherlands; 10 Department of Special Gynecology, Medical University of Vienna, Borschkegasse 8a, A-1090 Vienna, Austria

## Abstract

**Introduction:**

The Livial Intervention Following Breast Cancer: Efficacy, Recurrence and Tolerability Endpoints (LIBERATE: Clinical http://Trials.gov number NCT00408863), a randomized, placebo-controlled, double-blind trial that demonstrated that tibolone (Livial), a tissue-selective hormone-replacement therapy (HRT), increased breast cancer (BC) recurrence HR 1.40 (95% CI, 1.14 to 1.70; *P = 0.001*). A subgroup of women was entered into a study of bone mineral density (BMD).

**Methods:**

Women with surgically excised primary BC (T1-3, N0-2, M-0) within the last 5 years, complaining of vasomotor symptoms, were assigned to tibolone, 2.5 mg daily, or placebo treatment for a maximum of 5 years. The BMD substudy enrolled 763 patients, using dual-energy X-ray absorptiometry (DXA) scanning at baseline and at 2 years.

**Results:**

In the bone substudy, 699 of 763 women were eligible (345 allocated to tibolone, and 354, to placebo). After undergoing DXA scans, 300 (43%) women had normal BMD; 317 (45%), osteopenia; and 82 (11.7%), osteoporosis. Low body-mass index (*P *< 0.001), Asian race (*P < 0.001*), and late age at menarche (*P *< 0.04) predicted low bone mass at baseline. Tibolone increased BMD by 3.2% at the lumbar spine and 2.9% at the hip compared with placebo (both *P < 0.001*). The majority of fractures (55%) occurred in osteopenic patients. Women with normal BMD had increased recurrence with tibolone, 22 (15.6%) of 141 compared with placebo, 11 (6.9%) of 159 (*P = 0.016)*, whereas no increased BC recurrence was seen in women with low BMD; 15 (7.4%) of 204 taking tibolone versus 13 (6.7%) of 195 taking placebo.

**Conclusions:**

Tibolone is contraindicated after BC treatment, as it increases BMD and BC recurrence. Risk of BC recurrence was elevated in BC women with normal BMD (compared with low) who took tibolone.

## Introduction

Osteoporosis (reduced bone mineral density (BMD)) leads to fractures that severely affect the quality of life [1]. Postmenopausal women have increased bone loss due to estrogen deficiency, resulting in an increased fracture risk. Fracture risk also increases after a diagnosis of breast cancer [1,2]. Breast cancer (BC) patients frequently have accelerated bone loss because of chemotherapy, inducing premature menopause or aromatase inhibitor (AI) therapy, lowering estrogen levels, thus increasing fracture rate [3,4].

Although dual-energy X-ray absorptiometry (DXA) is proposed to identify those with low BMD in women commencing therapy, the incidence and frequency of osteoporosis in BC patients has not been widely studied. The bone substudies of the AI trials contained small numbers of patients [5,6].

Tibolone (Livial) is a synthetic steroid with a pharmacologic and clinical profile different from conventional sex steroids; it reduces vasomotor symptoms and prevents osteoporosis [7]. In the Longterm Intervention on Fractures with Tibolone (LIFT) trial, tibolone, 1.25 mg/day, prevented spinal fractures in osteoporotic older women compared with placebo, reducing the risk of BC (HR, 0.32; 0.13 to 0.80) [8]. Many women undergoing adjuvant therapy for BC have vasomotor symptoms such as hot flushes; both osteoporosis and vasomotor symptoms can potentially be prevented by the use of tibolone.

The Livial Intervention Following Breast Cancer; Efficacy, Recurrence and Tolerability Endpoints (LIBERATE) study [9] set out to demonstrate noninferiority of tibolone compared with placebo on BC recurrence, but closed early because of increased BC recurrence with tibolone.

Studies have suggested that normal BMD is associated with an increased risk of BC development [10,11]. The LIBERATE bone substudy, therefore, assessed the changes in BMD with tibolone and determined the relation between the effects on BMD and BC recurrence in this population.

## Materials and methods

LIBERATE (http://ClinicalTrials.gov number NCT00408863) was a randomized, placebo-controlled, double-blind, parallel-group trial of tibolone (Livial), 2.5 mg/day, on BC recurrence, aiming to demonstrate the noninferiority of treatment compared with placebo in women with climacteric symptoms and a history of BC [9].

The primary end point was BC recurrence rate. Secondary study outcomes included vasomotor symptoms, health-related quality of life (HRQL), overall survival, and BMD. In total, 3,583 women were screened, of whom 3,148 were randomized in 245 centers in 31 countries: 1,579 to tibolone and 1,569 to placebo [9].

**Figure 1:**
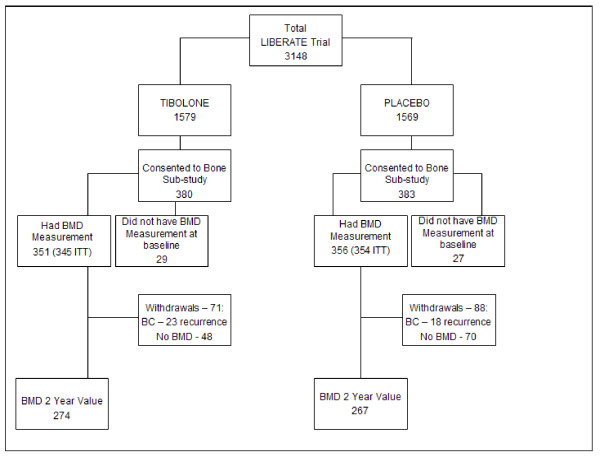
**CONSORT diagram of participant flow**.

The BMD substudy used DXA scanning at baseline and after 2 years or at trial discontinuation, as long as on trial medication. The aim was to explore the effect of tibolone compared with placebo on BMD of the lumbar vertebrae (L1 to L4) and left proximal femur for hip density. Of 763 women randomized to the BMD substudy, only 699 had BMD assessed at any site: 697 at the lumbar spine, and 691 at the hip, and entered the study (Figure 1).

BMD was measured by using Lunar or Hologic instruments. Bone densitometry data were acquired by DXA technicians, trained by the Quality Control/Quality Assurance (QC/QA) centers according to protocols prepared by central QC/QA facilities. These facilities were also responsible for continuous safety monitoring of incoming data and for complete QC/QA procedures, including cross-calibration of instruments at all clinical trial sites, and data analysis. This ensured comparability of the results over time and across different sites and machines. If a scan of the left femur was not possible, then the right femur could be used, and was used consistently throughout the trial. DXA scans were performed at baseline, after 2 years, or at trial discontinuation, as long as the patient remained taking the trial medication. Fractures were assessed by investigators reporting their presence as a serious adverse event.

### Patients

Women with histologically confirmed BC (T1-3, N0-2, M0), surgically treated within the last 5 years, irrespective of hormone-receptor status, were randomized between July 2002 and December 2004. Patients were younger than 75 years, with the last menstruation at least 12 months before study start or ovariectomized, hysterectomized, or taking gonadotropin-releasing hormone (GnRH) analogues and with vasomotor symptoms, either related to natural menopause or resulting from prior or current adjuvant BC treatment. Use of tamoxifen, aromatase inhibitors, GnRH analogues, or chemotherapy was allowed. Recent or current use of estrogenic or progesterogenic substances, as well as any nonregistered investigational drug or Raloxifene hydrochloride, was not allowed. Bisphosphonates were not allowed before study entry, and only 7% of women ever used bisphosphonates in the LIBERATE trial. All women gave informed consent, and the study was approved by the Ethics Committees and/or Health Authorities of the hospitals and countries involved.

### Statistical analysis

All analyses were carried out for 699 women with a BMD value at baseline in the intent-to-treat (ITT) group. To deal with drop-outs, the observed case approach was used. The following populations were considered: overall population, Caucasian, and Asian race.

According to the World Health Organization (WHO) criteria, BMD was divided into three categories based on the T-scores in the total hip: osteoporosis if T-score in at least one site of 2.5 or less; osteopenia if T-score in both sites was more than or equal to 2.5, and at least for one site, the T-score was 1 or less; and normal if the T-score in both sites was 1 or more [1].

For lumbar spine and total hip, multiple regression analysis with forward stepwise selection was performed to identify predicting factors for BMD at baseline as a continuous variable. Prognostic factors were further examined by fitting a logistic regression by using the BMD classes osteoporotic and not normal (osteopenic and osteoporotic) as response variables.

For each of the sites, change from baseline in BMD and percentage change from baseline in BMD were analyzed by using an analysis of covariance (ANCOVA) model with, as factors, Treatment group and Center, and as covariate, the Baseline value. Additionally, both binary outcomes, osteopenia and osteoporosis, were analyzed by using logistic regression models with, as factors, Treatment group, Race, and as a covariate, BMI.

Differences in bone loss (defined as any decrease in BMD from baseline) at both sites between tibolone and placebo groups were analyzed by using the Pearson χ^2 ^test.

The occurrence of fractures was analyzed by using a logistic regression model with, as factors, Treatment group, Race (Caucasian versus Asian), Treatment by race interaction, and BMD classes (osteoporotic versus normal and osteopenic versus normal), and as covariates, Age, Baseline body mass index (BMI), and Age at menarche (and/or menopause).

Time to BC recurrence was analyzed by using a Cox proportional hazards model, stratified by country, with factors for Treatment and BMD classes and a covariate for baseline BMI. A similar model is fitted with BMD class (osteopenic and osteoporotic grouped into one class) as a time-dependent factor to account for updated BMD information during the first 2 years after randomization.

To examine a linear trend with regard to the effect of BMD classes on BC recurrence in the tibolone group and the placebo group, similar models using BMD classes as a continuous variable were fitted to each treatment group separately, and the consistency of the linear trend across the treatment groups was tested.

### Role of funding source

An Independent International Steering Committee advised on trial safety and conduct. The trial was funded by Schering Plough.

## Results

As previously reported, tibolone increased BC relapse rates HR, 1.4 (95% CI, 1.14 to 1.70; *P *= 0.001), and the trial closed prematurely [9].

Demographics and baseline characteristics of 699 subjects who consented to the bone substudy and had a BMD assessment at baseline (on or before day 1) of either site are presented in Table 1 and Figure 1.

**Table 1:** Demographics and other baseline characteristics of subjects with BMD data at baseline

			Tibolone 2.5 mg (*n *= 345)			Placebo (*n *= 354)		
			Osteoporosis (*n *= 39)	Osteopenia (*n *= 165)	Normal (*n *= 141)	Osteoporosis (*n *= 43)	Osteopenia (*n *= 152)	Normal (*n *= 159)
**Age (years)**								
		Mean (SD)	55.3 (7.3)	53.1 (7.3)	52.9 (7.4)	56.8 (7.2)	53.3 (7.0)	52.2 (6.4)
		Median (range)	55.0 (43, 73)	53.0 (32, 74)	53.0 (34, 72)	57.0 (42, 70)	53.0 (36, 71)	52.0 (39, 75)
**Body mass index (kg/m^2^)**^a^		N	36	164	138	43	151	158
		Mean (SD)	24.0 (3.5)	25.1 (4.6)	26.5 (3.8)	24.3 (4.0)	25.1 (4.1)	27.7 (4.8)
		Median (range)	23.9 (18, 33)	23.8 (17, 52)	25.7 (19, 37)	24.0 (19, 37)	23.9 (19, 39)	26.9 (19, 48)
**Time since menopause (years)**^a^		N	35	143	116	39	135	134
		Mean (SD)	10.5 (7.9)	6.9 (6.5)	7.3 (7.4)	8.4 (6.4)	7.1 (7.1)	5.0 (5.3)
		Median (range)	8.4 (1, 35)	4.3 (0, 30)	4.6 (0, 31)	5.1 (0, 24)	4.3 (1, 30)	3.1 (0, 30)
**Time since breast cancer surgery (years)**		N	39	165	141	43	152	159
		Mean (SD)	2.4 (1.2)	2.2 (1.2)	2.2 (1.3)	2.4 (1.5)	2.3 (1.3)	2.0 (1.2)
		Median (range)	2.3 (0, 5)	2.0 (0, 5)	2.1 (0, 5)	2.4 (0, 5)	2.1 (0, 5)	1.7 (0, 5)
**Race**	***n *(%)**	Asian	26 (66.7)	76 (46.1)	31 (22.0)	26 (60.5)	63 (41.4)	38 (23.9)
		Black					1 (0.7)	1 (0.6)
		Caucasian	13 (33.3)	85 (51.5)	109 (77.3)	16 (37.2)	86 (56.6)	118 (74.2)
		Other		4 (2.4)	1 (0.7)	1 (2.3)	2 (1.3)	2 (1.3)
**Smoking at baseline**	***n *(%)**	No	39 (100.0)	144 (87.3)	122 (86.5)	38 (88.4)	133 (87.5)	137 (86.2)
		Yes		21 (12.7)	19 (13.5)	5 (11.6)	19 (12.5)	22 (13.8)
**Alcohol use at baseline**	***n *(%)**	No	36 (92.3)	111 (67.3)	78 (55.3)	32 (74.4)	96 (63.2)	83 (52.2)
		Yes	3 (7.7)	54 (32.7)	63 (44.7)	11 (25.6)	56 (36.8)	76 (47.8)
**Estrogen-receptor status**	*n *(%)	Negative	12 (30.8)	36 (21.8)	21 (14.9)	12 (27.9)	49 (32.2)	30 (18.9)
		Positive	27 (69.2)	122 (73.9)	116 (82.3)	29 (67.4)	101 (66.4)	126 (79.2)
		Unknown		7 (4.2)	4 (2.8)	2 (4.7)	2 (1.3)	3 (1.9)
**Progestagen-receptor status**	*n *(%)	Negative	11 (28.2)	45 (27.3)	28 (19.9)	16 (37.2)	58 (38.2)	39 (24.5)
		Positive	27 (69.2)	110 (66.7)	106 (75.2)	25 (58.1)	86 (56.6)	111 (69.8)
		Unknown	1 (2.6)	10 (6.1)	7 (5.0)	2 (4.7)	8 (5.3)	9 (5.7)
**Aromatase Inhibitor**^a^	*n *(%)	None	33 (84.6)	149 (90.3)	132 (93.6)	39 (90.7)	139 (91.4)	144 (90.6)
		Ever, but not recent	1 (2.6)	3 (1.8)		1 (2.3)	4 (2.6)	2 (1.3)
		Recent	5 (12.8)	13 (7.9)	9 (6.4)	3 (7.0)	9 (5.9)	13 (8.2)
**Tamoxifen**^a^	*n *(%)	None	13 (33.3)	36 (21.8)	29 (20.6)	15 (34.9)	48 (31.6)	29 (18.2)
		Ever, but not recent	4 (10.3)	16 (9.7)	9 (6.4)	1 (2.3)	10 (6.6)	14 (8.8)
		Recent	22 (56.4)	113 (68.5)	103 (73.0)	27 (62.8)	94 (61.8)	116 (73.0)
GnRH analogues^a^	*n *(%)	None	34 (87.2)	159 (96.4)	133 (94.3)	41 (95.3)	144 (94.7)	147 (92.5)
		Ever, but not recent	1 (2.6)	1 (0.6)	2 (1.4)	1 (2.3)	3 (2.0)	1 (0.6)
		Recent	4 (10.3)	5 (3.0)	6 (4.3)	1 (2.3)	5 (3.3)	11 (6.9)
Chemotherapy^a^	*n *(%)	None	11 (28.2)	52 (31.5)	54 (38.3)	12 (27.9)	55 (36.2)	59 (37.1)
		Ever, but not recent	25 (64.1)	109 (66.1)	83 (58.9)	28 (65.1)	94 (61.8)	96 (60.4)
		Recent	3 (7.7)	4 (2.4)	4 (2.8)	3 (7.0)	3 (2.0)	4 (2.5)
Node status	*n *(%)	Missing					1 (0.7)	
		Negative	18 (46.2)	90 (54.5)	86 (61.0)	20 (46.5)	80 (52.6)	93 (58.5)
		Positive	21 (53.8)	75 (45.5)	55 (39.0)	23 (53.5)	71 (46.7)	66 (41.5)

^a^Statistical analysis revealed no differences between groups for all of the above demographics and other baseline characteristics. Body mass index (*P *= 0.06) and time since menopause (*P *= 0.06) were the only two continuous variables close to significance. Comparison between tibolone and placebo randomized patients at baseline demonstrated no difference between groups for all of the demographics and other baseline characteristics. Body Mass Index (*P *= 0.06) and time since menopause (*P *= 0.06) were the only two continuous variables that came close to significance.

**Figure 2:**
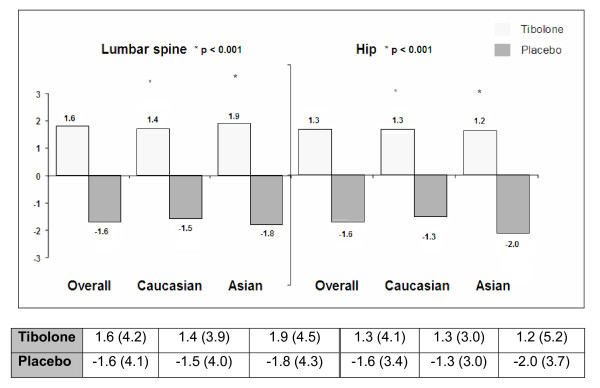
**Bone mineral density change (%) from baseline after 2 years**. Figure shows relative change from baseline of BMD (mean (SD)). BMD changes on tibolone compared with placebo between baseline and 2 years of treatment. Tibolone significantly increased BMD, whereas patients taking placebo had a 2% loss of BMD and had a lower weight and height. Overall, a 1.6% increase was found in BMD at lumbar spine on tibolone and a 1.6% BMD loss on placebo, and similar magnitudes of change were seen at the hip (both *P *≤ 0.01).

For both sites, (lumbar spine (LS) and hip), the tibolone group showed an absolute increase in BMD after 2 years of treatment compared with an absolute decrease in BMD seen with placebo (Figure 2/Additional file 1 Table S1: *P *< 0.001).

The percentage change from baseline for LS BMD and total hip was 1.6% and 1.3%, respectively, in the tibolone group, with a decrease of 1.6% at both sites observed taking placebo (Figure 2 and Additional file 1 Table S1).

The percentage changes from baseline adjusted treatment effects were 3.12 (95% CI, 2.41 to 3.84) and 2.85 (95% CI, 2.20 to 3.49) for the LS and total hip, respectively. The Asian and Caucasian subgroups were similar at both the LS (*P *= 0.65) and total hip (*P *= 0.14).

### Prediction of osteoporosis at baseline and at treatment

At baseline, 697 subjects had information on BMD of LS (343 in the tibolone group versus 354 in the placebo group), and 691 subjects had information on BMD of the total hip (342 in the tibolone group versus 349 in the placebo group). The majority of subjects were of Asian (37.2%) or Caucasian (61.1%) races. Overall, 82 (11.7%) had osteoporosis in either hip or LS, 317 (45.4%) had osteopenia, and 300 (42.9%) had normal BMD in both sites. Asian women (63.4%) contributed the majority of women with osteoporosis, followed by Caucasian women (35.4%). The osteopenia group consisted of 53.9% Caucasians and 43.9% Asians. The distribution of BMD categories based on T-score at both sites was comparable among the treatment groups, as were demographics and baseline characteristics (Table 1) [12].

However, Asian women were more likely to be osteoporotic or osteopenic (*P *< 0.0001). Asian women had lower weight and height (wt, 59.2 kg; ht, 156 cm) compared with their Caucasian counterparts (wt, 71.5 kg; ht, 164 cm); both *P *< 0.0001.

After a stepwise selection procedure, Asian race, older age, late age at menarche, longer time since breast surgery, and low BMI were found to be significant (all *P *values < 0.05) with regard to both total hip and LS T-score at baseline (Table 2) and predicted nonnormal BMD class (T-score 1 or less at both sites), as well as osteoporosis (T-score, 2.5 or less) at baseline. Medical oophorectomy by GnRH analogue use also predicted LS but not total hip T-score at baseline (*P *= 0.039).

**Table 2:** Factors predicting bone mineral density (lumbar spine and total hip)

		Numerator	Denominator		
End point	Effect	DF	DF	*F *value	*P *value
Lumbar spine	Race	2	665	26.19	< 0.0001
	BMI	1	665	17.06	< 0.0001
	Time since breast surgery (years)	1	665	7.74	0.006
	AGE	1	665	6.32	0.012
	Age at menarche (years)	1	665	4.74	0.030
	Node status	1	665	4.77	0.029
	GnRH analogues	2	665	3.26	0.039
Total hip	Race	2	660	63.33	< 0.0001
	BMI	1	660	149.15	< 0.0001
	Time since breast surgery (years)	1	660	6.15	0.013
	AGE	1	660	4.81	0.029

Factors predicting bone mineral density (BMD) at baseline. Asian women show significantly more osteoporosis (*P *≤ 0.001) at both hip and lumbar spine. Older age and lower BMI, both of which are recognized to predict BMD, were also significant.

At baseline, 11.7% of subjects were osteoporotic, but after 2 years of treatment, 15.4% in the placebo group and 10.2% in the tibolone group remained osteoporotic. In addition to osteoporosis at baseline, factors predicting osteoporosis after 2 years were BMI (OR, 0.87; 95% CI, 0.78 to 0.98; *P *= 0.019), and treatment (tibolone versus placebo, OR, 0.38; 95% CI, 0.16 to 0.88; *P *= 0.024). For nonnormal BMD class, ethnicity was also an additional risk factor (Asian race versus Caucasian race, OR, 3.02; 95% CI, 1.61 to 5.68; *P *= 0.0006).

For LS, the number of subjects who experienced bone loss was 99 (37.2%) with tibolone versus 174 (66.4%) with placebo (LS, *P *< 0.0001), whereas total hip-bone loss occurred in 86 (32.0%) women taking tibolone versus 176 (66.9%) in the placebo group (*P *< 0.0001).

### BMD and fractures

Thirty-eight fractures occurred, of which the majority, 21, occurred in the osteopenic group, 12 in the normal BMD group, and only five in the osteoporotic group (Table 3). Fractures occurred in 6.8% of osteopenic, 6.2% of osteoporotic, and 4.1% of normal-BMD women. Logistic regression analysis, including age, BMI, age at menarche and/or menopause, BMD classes at baseline, treatment, race, and Treatment-by-Race interaction, and race found no significant predictors of fracture (data not shown). No significant effect of tibolone on fracture rate was found, although in the Caucasian women, seven of 206 taking tibolone developed fractures compared with 17 of 221 taking placebo (χ^2^, *P *= 0.054). Testing homogeneity of the tibolone-to-placebo odds ratios revealed no difference between the Asian and Caucasian races, as indicated by lack of Treatment-by-Race interaction.

**Table 3:** Fractures by BMD classification at baseline (on or before day 1), Asian and Caucasian

		Tibolone 2.5 mg			Placebo			All		
		*n*	Number of fractures	%	*n*	Number of fractures	%	*n*	Number of fractures	%
**Asian**	All	133	6	4.5	127	8	6.3	260	14	5.4
	Osteoporosis	27	1	3.7	25	2	8.0	52	3	5.8
	Osteopenia	75	4	5.3	64	5	7.8	139	9	6.5
	Normal	31	1	3.2	38	1	2.6	69	2	2.9
**Caucasian**	All	206	7	3.4	221	17	7.7	427	24	5.6
	Osteoporosis	12	0	0.0	17	2	11.8	29	2	6.9
	Osteopenia	85	4	4.7	86	8	9.3	171	12	7.0
	Normal	109	3	2.8	118	7	5.9	227	10	4.4
**All**	All	339	13	3.8	348	25	7.2	687	38	5.5
	Osteoporosis	39	1	2.6	42	4	9.5	81	5	6.2
	Osteopenia	160	8	5.0	150	13	8.7	310	21	6.8
	Normal	140	4	2.9	156	8	5.1	296	12	4.1

No significant reduction in fractures occurred in the group overall, although an almost-significant reduction occurred in Caucasian patients taking tibolone (p = 0.054). Fractures according to baseline bone mineral density (BMD) classification: The percentage of women who developed bone fractures did not differ between those with osteoporosis and osteopenia, but because osteopenia was the larger population group, more fractures occurred among osteopenic patients.

### Analysis of BC recurrence restricted to subjects with BMD data

**Figure 3:**
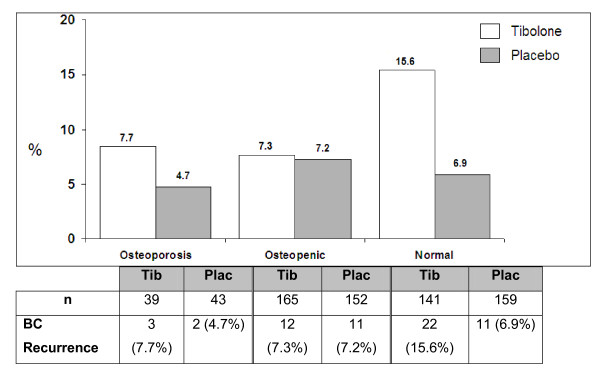
**Baseline bone mineral density (BMD) and breast cancer recurrence**. Incidence of breast cancer recurrence with tibolone or placebo according to baseline BMD. Significantly more breast cancers occurred in women with normal BMD taking tibolone (15.6%) compared with either osteopenic or osteoporotic patients (7.3% and 7.7%, respectively; *P *≤ 0.016).

For subjects with BMD data, 61 women have experienced a BC recurrence. Univariate analysis of the effect of tibolone treatment on inducing BC recurrence according to BMD at baseline, grouped as osteoporosis, osteopenia, or normal, revealed recurrence with placebo was 4.7%, 7.2%, and 6.9%, respectively, whereas for tibolone, it was 7.5%, 7.3%, and increased to 15.6% in women with normal BMD (*P *= 0.03; Figure 3 and Additional file 2 Table S2).

Bisphosphonate use did not affect time to BC recurrence (HR, 0.82 (95% CI, 0.53 to 1.27)).

Results of fitting a proportional hazard Cox model with Treatment, Osteopenia status, and Osteoporosis status as factors and BMI as covariate indicate that tibolone and normal BMD increased the BC recurrence compared with placebo and osteopenia, respectively. Fitting the same model for tibolone and placebo groups separately suggested an increase in BC recurrence in patients, with an increase in BMD seen in tibolone group (HR = 0.47; 95% CI, 0.26 to 0.85; *P *= 0.017; Additional file 3 Table S3) but not in the placebo group (HR, 0.89; 95% CI, 0.47 to 1.68; *P *= 0.65), although testing the homogeneity of the linear trend over BMD classes indicated no differences between treatment groups (*P *= 0.19).

Table 4 groups osteopenic and osteoporotic patients together and uses this as a time-dependent covariate to correct for changes in BMD during the course of the first 2 years of the trial. The results indicate again that tibolone and normal BMD are associated with increased BC recurrence.

**Table 4:** Analysis of variance table for breast cancer recurrence

Parameter	Estimate	Std error	Χ^2^	DF	*P *value	Hazard ratio	95% Confidence limits
Tibolone	0.50	0.26	3.75	1	0.05	1.64	0.99, 2.72
BMI	-0.01	0.03	0.07	1	0.79	0.99	0.93, 1.06
Osteopenia (T-score ≤ -1)	-0.57	0.27	4.41	1	0.04	0.57	0.34, 0.96

These results are restricted to subjects with any BMD assessment, using a proportional hazard Cox model with Treatment, Osteopenia (defined as T-score ≤ -1) as time-dependent factors and Body mass index (BMI) as covariate. For those patients with baseline and either 2-year or second BMD measurements at trial end, an analysis was carried out to correct for changes in BMD on treatment. Osteopenic and osteoporotic patients were grouped together to take account of changes in BMD during the course of the trial. Tibolone use in women with normal BMD values was associated with increased breast cancer recurrence with a Hazard Ratio that reached significance.

## Discussion

Treatment of BC survivors with tibolone (a specific tissue estrogenic-activity regulator) led to increases in BMD and BC recurrence. Tibolone and other HRTs are contraindicated after breast cancer treatment.

Recurrence with tibolone occurred mainly in women with normal BMD. Osteoporosis increases with age, current smoking, and low BMI [1,2,12]. The reduction of estrogen levels increases BMD loss after the menopause and increases the risk of fracture [2-6].

In BC survivors, adjuvant therapies increase bone loss, leading to increased fractures and lower health-related quality of life [2]. The prevalence of osteoporosis has been reported to be up to 27% in BC survivors [11]; 11.7% of women recruited to the LIBERATE bone substudy were osteoporotic, and 45.4%, osteopenic, despite being only an average of 2.2 years since diagnosis.

The higher incidence of low BMD measurements (compared with the general population) and increased fracture rate observed in women after a diagnosis of BC [2] has resulted in ASCO and European Guidelines suggesting that all women diagnosed with BC should undergo DXA scanning to detect those women at risk of fracture [7] and to initiate treatment at an early stage if osteoporosis is indicated by the DXA results.

BMD scanning identifies women with BC at risk of fragility fracture, but 55% of fractures occurred in the osteopenic group compared with 13% among the "high risk" osteoporotic women (Table 3), indicating that BMD alone cannot be used to select patients for antiresorptive therapy to prevent fragility fracture. Few (6.5%) patients in this study were taking AI, but the fracture rate (2.8% per annum) remains comparable with that reported in patients taking AIs in randomized trials [3-6]. Treatment with intravenous zoledronic acid, 4 mg/day, has been shown to increase BMD in pre- and postmenopausal women with breast cancer [13], and recent results from the ABCSG-12/ZO-FAST trials suggest that when combined with endocrine therapy, this combination improved disease-free recurrence compared with endocrine therapy alone, but the Zoledronic Acid Adjuvant Therapy (AZURE) trial results did not confirm a disease-recurrence benefit. However, bisphosphonates (not hormone-replacement therapies) should be used in BC survivors with low BMD [13-15].

Current American Society of Clinical Oncology and UK guidelines recommend bisphosphonate therapy for women with a T score of 2.0 or more. To prevent the majority of fractures would require all women with T scores greater than -1.0 to undergo antiresorptive therapy [7]. An alternative approach in osteopenic women with BC advocates commencing antiresorptive therapy in women with one other recognized risk factor for fracture, such as history of fragility fracture, BMI < 20, corticosteroid use, or a cigarette-smoking habit [16].

BMD is a function of the lifetime exposure of a woman to estrogen [12,17]. In the LIBERATE study, osteoporosis was associated with older age, lower BMI, late age at menarche, and Asian race. Important racial differences in BMD exist, with lower BMD in the Asian race compared with Caucasians. The application of Caucasian reference values to an Asian population may not reflect the true osteoporosis rate and fracture risk in Asians [17,18]. After adjusting for other factors, such as height and weight, the BMD and bone mass are reported not to differ between Asians and Caucasians [19]. However, trabecular BMD decreases at an earlier age in Asian women, and the fracture threshold (especially spinal fracture) is lower [20]. Fracture rates in the Asian population were comparable to those of Caucasians, indicating that a low BMD in Asian women is predictive of fracture risk.

In the LIFT trial, tibolone reduced fractures in osteoporotic women [8], and in the LIBERATE Caucasian population, tibolone increased BMD and reduced fracture risk (OR = 0.42; *P *= 0.06). Tibolone increased BMD regardless of ethnic background.

### Breast cancer risk

BC risk is associated with a high lifetime exposure to estrogen, and BMD may be a surrogate marker for this exposure. Studies have shown an association between higher BMD and increased risk of BC [11-13,15]. The Women's Health Initiative study indicated that hip BMD predicted BC risk independent of Gail score [21]. The contribution of BMD in the prediction of postmenopausal BC score was significant in a Cox proportional hazards model and independent of the Gail score. Normal BMD appears to be associated with an increased BC risk [22].

In the Multiple Outcomes of Raloxifene Evaluation (MORE) trial, women with low bone mass (T-score, 2 or less) had a higher incidence of invasive, estrogen receptor-positive BC than did those with osteoporosis (HR, 2.13; 95% CI, 1.12 to 4.03). Raloxifene, an antiestrogen, reduced the incidence of BC development in this group [11]. In the LIFT trial, tibolone (1.25 mg/day) likewise reduced BC risk in a population of osteoporotic women [8].

In the LIBERATE study, which used tibolone, 2.5 mg/day, in women after BC treatment, the incidence of BC recurrence increased. Recurrence was highest in the group with normal BMD randomized to tibolone compared with those with low BMD. In the placebo group, an increase in BC recurrence was seen in the normal and osteopenic women, compared with osteoporotic women. The finding that normal BMD is associated with increased BC recurrence provides support to the concept that normal BMD is a surrogate for postmenopausal estrogen levels or that women with normal BMD are more sensitive to estrogenic stimulation. BMD may be a biomarker of drug effect, so that as BMD gain occurred with tibolone, BC risk increased (Table 4). This is similar to the reduced BC risk seen in the MORE Trial, with increased BMD gain with the antiestrogen raloxifene. Alternatively, women with higher BMD may harbor single-nucleotide polymorphisms (SNPs) of the estrogen-receptor alpha or *CYP 17/19 *genes, which result in higher ER alpha at similar estrogen levels and reduce the risk of developing early osteoporosis and fracture [23]. Follow-up of women with known BMD and ER SNPs should allow this possibility to be explored.

## Conclusions

Women with normal bone density not only have a higher lifetime exposure to estrogen but also have a lower threshold for estrogen stimulation of BMD increases and BC recrudescence. The placebo groups in studies protecting against AI-induced bone loss by using bisphosphonates will allow confirmation (or not) that low BMD is associated with a low risk of BC recurrence.

Tibolone and other HRTs should not be used as osteoporosis treatment in women with breast cancer, as they increase the risk of recurrence.

## Abbreviations

AI: aromatase inhibitor; ANCOVA: analysis of covariance; BC: breast cancer; BMD: bone mineral density; BMI: body mass index; DXA: dual-energy X-ray absorptiometry; HRQL: health-related quality of life; HRT: hormone-replacement therapy; ITT: intent-to-treat; LIBERATE: Livial Intervention Following Breast Cancer; Efficacy, Recurrence and Tolerability Endpoints; LIFT: Longterm Intervention on Fractures with Tibolone; LS: lumbar spine; MORE: Multiple Outcomes of Raloxifene Evaluation; QA: quality assurance; QC: quality control; SNPs: single-nucleotide polymorphisms; WHO: World Health Organization.

## Competing interests

PK, NJB, JMF, EK, BvS, PS, RVS, MWB, and CHY have received honoraria for their membership on the LIBERATE Advisory Board. NJB was a member of the LIBERATE Steering Committee and has received fees for lecturing on tibolone. Schering-Plough is now owned by Merck.

PK has received research grants and honoraria for consultancies from the following pharmaceutical companies: Schering-Plough, Procter & Gamble, Servier, and Pfizer.

NJB has received honoraria for consultancies and postgraduate education lectures from Schering-Plough and has served on advisory boards for Schering-Plough, Astra-Zeneca, Novartis, and Pfizer.

MWB has served on advisory boards for GSK, Novartis, Astra-Zeneca, Sanofi, Aventis, and Schering-Plough.

JE, RM, MM-A, and SvO are employees of Schering-Plough (formerly NV Organon).

## Authors' contributions

All authors read and approved this manuscript for publication. All authors are members of the Scientific Advisory Board or employees of Schering-Plough Corporation and contributed to the study concept, design, and implementation, and to content and development of this manuscript.

## LIBERATE study group

Advisory Board: P Kenemans (The Netherlands), JM Foidart (Belgium), NJ Bundred (UK), E Kubista (Austria), B von Schoultz (Sweden), P Sismondi (Italy), R Vassilopoulou-Sellin (USA), M Beckmann (Germany), and CH Yip (Malaysia).

Independent DSMB: M Baum (UK), R Gray (UK) and CW Burger (The Netherlands).

Breast & Gynaecological Cancer Adjudication Committee: P.J. van Diest (The Netherlands), N. Harbeck (Germany) and R. Gray (UK)

Cardiovascular Adjudication Committee: MH Prins (The Netherlands), B. Davidson USA), R. Peters (The Netherlands), ML Longo (USA) and LJ Kappelle (The Netherlands)

Investigators: AUSTRALIA: R Baber, JA Eden, C Furnival, B Stuckey, E Farrell and AH MacLennan. AUSTRIA: CF Singer, E Kubista, C Marth, R Reitsamer, P Sevelda, H Salzer, I Thiel and R Winter. BELARUS: L Putyrski, V Belyakovski and Gedrevich. BELGIUM: W Tjalma, J Desreux, M Dhont, H Depypere, R van den Broecke, JM Foidart, M L'Hermite, JP Nolens, S Rozenberg, Ph Simon, I Vergote, U Gaspard, D Janssens, H De Gezelle and E Merchiers. BRAZIL: JM Aldrighi, M Badalotti, VR Bagnoli, CE Fernandes, R Ferriani, AM Pinto Neto, CH Menke, DA Petti, A Urbanetz and A Del Giglio. CHILE: Cunill, H Sepulveda, L Soto, A Uribe and I Valdivia. COSTA RICA: J Baptista and K Loaciga. CZECH REPUBLIC: M Bendova, K Buchta, V Pecha, T Reslova, O Hlavackova and M Mikulik. ESTONIA: R. Kütner and P Padrik. FINLAND: U Puistola, O Ylikorkala and K Mäenpää-Liukko. FRANCE: A Brémond, C Faure, P Seffert, T Delozier, M Espie, D Dupaigne, M Hoffet, F Laffargue, C Tunon de Lara, L Largillier, M Namer, L Dognon, T Routiot, P This and P Touraine. GERMANY: R Kimmig, S Kümmel, M Beckmann, J Weiss, C Sohn, G Bastert, C Nestle-Krämling, B Blümel, U Engel, C Brucker, D Chatsiproios, B Conrad, HJ Hindenburg, C Höss, W Jonat, L Kiesel, B Krause-Bergmann, T Dewitz, T Kühn, U Köhler, R Landthaler, D Langanke, H Leitsmann Thomas, W Lichtenegger, P Mallmann, Neumann, Kulp, O Ortmann, C Schindler, P Schmidt-Rhode, W Schoenegg, V Seifert-Klauss, G Splitt, F Starflinger, I Rühl, M Untch, JC de Waal, D Wallwiener and R de Wilde. HUNGARY: M Baki, Boér, J Boros, J Cseh, Faluhelvi, E Kövér, T Nagykálnai, A Ruzsa, J Erfán, F Fábián and K Páli. ITALY: AR Genazzani, S Ricci, R Mariani, A Martoni, G Scambia, N Biglia, P Sismondi, A Santoro, S Burlizzi, G Amunni and D Amadori. KOREA: DY Noh, JG Kim, SH Ahn, BM Kang, WC Noh, MH Kim, MH Lee and JJ Lee. LATVIA: G. Keire and D Baltina. MALAYSIA: N Nik N Nasri, P Gomez, S Sivalingam, S Muniandy and CH Yip. MEXICO: J Cardenas, F Mainero, S Uscanga, A Fuentes and R Lugo. NETHERLANDS: H Heijmans, HS The, H Franke, J van Riel, S van der Vegt, E Boven, P Houben, A Kok, H van Weering, A van de Walle, Burggraaf and R Mulder. PANAMA: J. Alcedo. POLAND: J. Kornafel, M Litwiniuk, H Karnicka, L Bablok, Marianowski, A Basta and A Jakimiuk. ROMANIA: S. Curescu, ME Draganescu, AE Eniu, E Zbranca, G Peltecu, and V Ancar. RUSSIA: V Smetnik, A Petrossian, O Stekolschikova, A Popov, N Zoziouk, LI Krikunova, V Semiglazov, M Konstantinova, VF Bezhenar, A Diatchouk, G Manikhas, LY Korytova, V Vinogradov, V Sokurenko, Baranov, V.L. Arkhipovsky, IK Bogatova, LI Akhmadulina, Kuts, SA Susloparova, I Mitashok, AL Kanzaliev, VV Bryuzgin, EN Malygin, IE Sergeev, NM Pasman, Akishina, AV Tuev, EP Kullikov, M Kopp, VI Soloviev, ID Evtushenko, GV Ershov, NF Devyatchenko, YN Potapov, SV Cheporov, Ogurtsov, ON Chrustalev, DG Lazareva, E Bryukhina and M Matrosova. SINGAPORE: SL Yu and PC Wong. SLOVAK REPUBLIC: O Sadovsky, Z Petrovicova, L Masak and P Suska. SPAIN: JL. De Pablo, E García and J Iglesias. SWEDEN: L. Mattsson, G Granberg, L Berglund, and B Friberg. TAIWAN: ST Chen, SN Chow, JN Lee, KL Wang and TS Yang. THAILAND: KK Limpaphayom, S Boonjong, M Jirapinyo, C Sakondhavat, N Taechakraichana, K Techatraisak and K Wilawan. UKRAINE: L Dudar, Koshukova, V Gyoyachyy, Y Hotko, I Kostynskyy, V Paramonov, T Pertseva, Y Shparyk, T Tatarchuk, O Tarasova, N Kosey, Y Solskiy, I Smolanka, VP Kvashenko, O Grishyna and G Dzyak. UNITED KINGDOM: NJ. Bundred, JP Drew, R Mansel, J Williamson, CR Kingsland, L Vishwanath, R Bliss, D Crawford, Z Winters, D Browell, M Paterson, T Ind, A Rostom and J Pitkin.

## Supplementary Material

Additional file 1 **Summary statistics of the bone mineral density in the lumbar spine and total hip at baseline including change and percentage change from baseline**. Table showing the summary statistics of bone mineral density (BMD) change with tibolone or placebo from baseline at the lumbar spine and hip. This represents the raw data from Figure 2 and Additional file 2 Table S2. Analysis for breast cancer recurrence restricted subjects with any BMD assessment with a Proportional Hazard Cox model for Treatment and Osteopenia as time-dependent factors, with BMI as covariate. Click here for file

Additional file 2 **Analysis of variance table for BC recurrence restricted to subjects with any bone mineral density (BMD) assessment by using a proportional hazard Cox model with Treatment and Osteopenia (defined as T-score ≤ -1) as time-dependent factors and BMI as covariate**. This analysis groups osteopenic and osteoporotic patients together, as approximately 50% of patients had normal BMD, and uses a covariate to correct for changes in BMD during the course of the first 2 years of the trial. The results indicated that tibolone and normal BMD are associated with increased breast cancer recurrence. Click here for file

Additional file 3 **Breast cancer recurrence in the LIBERATE trial by body mass index (BMI), race, and lifestyle subgroups**. The effect of tibolone on breast cancer recurrence occurred in all races, although significance was reached in only Caucasians. Low or normal BMI was associated with increased breast cancer risk with tibolone (not high BMI). Click here for file
