# Supplementary material for: Tibolone increases bone mineral density but also relapse in breast cancer survivors: LIBERATE trial bone substudy
Source: Breast Cancer Res. 2012 Jan 17;14(1):R13. doi: 10.1186/bcr3097 (PMC3496130; doi:10.1186/bcr3097)
Supplement: Additional file 1 — Summary statistics of the bone mineral density in the lumbar spine and total hip at baseline including change and percentage change from baseline. Table showing the summary statistics of bone mineral density (BMD) change with tibolone or placebo from baseline at the lumbar spine and hip. This represents the raw data from Figure 2 and Additional file 2 Table S2. Analysis for breast cancer recurrence restricted subjects with any BMD assessment with a Proportional Hazard Cox model for Treatment and Osteopenia as time-dependent factors, with BMI as covariate. [file bcr3097-S1.DOC]

|  | | | **Treatment group** | | |
| --- | --- | --- | --- | --- | --- |
| **Parameter** | **Assessment** | | **Tibolone 2.5 mg (N=1556)** | **Placebo (N=1542)** | **P-value** |
| Lumbar spine  (gr/cm2) | Baseline | Value |  |  |  |
|  |  | n | 343 | 354 |  |
|  |  | Mean (SD) | 0.9900 (0.1541) | 1.0044 (0.1674) |  |
|  |  | Median (Min;Max) | 0.979 (0.591;1.424) | 1.009 (0.566;1.499) |  |
|  | Week 104 (yr 2) | Value |  |  |  |
|  |  | n | 266 | 262 |  |
|  |  | Mean (SD) | 0.9918 (0.1517) | 0.9849 (0.1707) |  |
|  |  | Median (Min;Max) | 0.982 (0.659;1.418) | 0.977 (0.580;1.524) |  |
|  |  | Change from baseline |  |  | <.001 |
|  |  | n | 266 | 262 |  |
|  |  | Mean (SD) | 0.0144 (0.0398) | -0.0170 (0.0403) |  |
|  |  | Median (Min;Max) | 0.011 (-0.093;0.128) | -0.019 (-0.123;0.100) |  |
|  |  | Relative change from baseline (%) |  |  | <.001 |
|  |  | n | 266 | 262 |  |
|  |  | Mean (SD) | 1.6 (4.2) | -1.6 (4.1) |  |
|  |  | Median (Min;Max) | 1.0 (-9.6;13.4) | -1.8 (-12.6;11.5) |  |
| Total hip (gr/cm2) | Baseline | Value |  |  |  |
|  |  | n | 342 | 349 |  |
|  |  | Mean (SD) | 0.9061 (0.1278) | 0.9167 (0.1372) |  |
|  |  | Median (Min;Max) | 0.902 (0.483;1.218) | 0.912 (0.600;1.298) |  |
|  | Week 104 (yr 2) | Value |  |  |  |
|  |  | n | 269 | 263 |  |
|  |  | Mean (SD) | 0.9131 (0.1311) | 0.8994 (0.1394) |  |
|  |  | Median (Min;Max) | 0.905 (0.494;1.247) | 0.897 (0.578;1.280) |  |
|  |  | Change from baseline |  |  | <.001 |
|  |  | n | 269 | 263 |  |
|  |  | Mean (SD) | 0.0107 (0.0367) | -0.0143 (0.0307) |  |
|  |  | Median (Min;Max) | 0.012 (-0.318;0.122) | -0.011 (-0.174;0.048) |  |
|  |  | Relative change from baseline (%) |  |  | <.001 |
|  |  | n | 269 | 263 |  |
|  |  | Mean (SD) | 1.3 (4.1) | -1.6 (3.4) |  |
|  |  | Median (Min;Max) | 1.4 (-33.4;15.7) | -1.2 (-21.7;5.4) |  |

P-value from ANCOVA model with treatment group and center terms as factors and baseline value as covariate.
