# Supplementary material for: Tibolone increases bone mineral density but also relapse in breast cancer survivors: LIBERATE trial bone substudy
Source: Breast Cancer Res. 2012 Jan 17;14(1):R13. doi: 10.1186/bcr3097 (PMC3496130; doi:10.1186/bcr3097)
Supplement: Additional file 2 — Analysis of variance table for BC recurrence restricted to subjects with any bone mineral density (BMD) assessment by using a proportional hazard Cox model with Treatment and Osteopenia (defined as T-score ≤ -1) as time-dependent factors and BMI as covariate. This analysis groups osteopenic and osteoporotic patients together, as approximately 50% of patients had normal BMD, and uses a covariate to correct for changes in BMD during the course of the first 2 years of the trial. The results indicated that tibolone and normal BMD are associated with increased breast cancer recurrence. [file bcr3097-S2.DOC]

| **Parameter** | **Estimate** | **Std Error** | **Chi-Square** | **DF** | **P-value** | **Hazard ratio** | **95 %**  **Confidence limits** |
| --- | --- | --- | --- | --- | --- | --- | --- |
| Tibolone | 0.50 | 0.26 | 3.75 | 1 | 0.05 | 1.64 | 0.99, 2.72 |
| BMI | -0.01 | 0.03 | 0.07 | 1 | 0.79 | 0.99 | 0.93, 1.06 |
| Osteopenia  (T-score≤ -1) | -0.57 | 0.27 | 4.41 | 1 | 0.04 | 0.57 | 0.34, 0.96 |
