# Supplementary material for: Tibolone increases bone mineral density but also relapse in breast cancer survivors: LIBERATE trial bone substudy
Source: Breast Cancer Res. 2012 Jan 17;14(1):R13. doi: 10.1186/bcr3097 (PMC3496130; doi:10.1186/bcr3097)
Supplement: Additional file 3 — Breast cancer recurrence in the LIBERATE trial by body mass index (BMI), race, and lifestyle subgroups. The effect of tibolone on breast cancer recurrence occurred in all races, although significance was reached in only Caucasians. Low or normal BMI was associated with increased breast cancer risk with tibolone (not high BMI). [file bcr3097-S3.DOC]

**Breast Cancer Recurrence (ITT) Hazard Ratio by Subgroup**

|  | **Tibolone**  **(N=1556)**  **N (5)** | **Placebo**  **(N=1542)**  **N (%)** | **HR [95% CI]** | **P-value** |
| --- | --- | --- | --- | --- |
| **Age (years)**   - **< 40** (n=88) - **40 – 49** (n=971) - **50 – 59** (n=1487) - **60 – 69** (n=509) | **14 (28.0%)**  **86 (17.1%)**  **106 (14.7%)**  **27 (10.2%)** | **8 (21.1%)**  **53 (11.3%)**  **77 (10.1%)**  **23 (9.4%)** | **1.28 [0.54;3.05]**  **1.56 [1.10;2.19]**  **1.45 [1.08;1.95]**  **1.06 [0.61;1.84]** | **NS**  **0.011**  **0.013**  **NS** |
| **BMI (kg/m2)**   - **< 25** (n=1213) - **25.0 – 29.9**  (n=1120) - **> 30** (n=747) | **78 (12.6%)**  **95 (16.8%)**  **63 (17.5%)** | **48 (8.1%)**  **63 (11.3%)**  **54 (14.0%)** | **1.55 [1.08;2.23]**  **1.48 [1.07;2.03]**  **1.24 [0.86;1.78]** | **0.016**  **0.015**  **NS** |
| **Race**   - **Asian** (n=536) - **Caucasian** (n=2452) - **Other** (n=95) | **31 (11.5%)**  **193 (15.7%)**  **10 (20.4%)** | **22 (8.3%)**  **136 (11.1%)**  **5 (10.9%)** | **1.39 [0.81;2.40]**  **1.40 [1.13;1.75]**  **1.84 [0.63;5.38]** | **NS**  **0.002**  **NS** |
| **Habits**   - **Smoking** (n=431) - **Alcohol use** (n=1042) | **40 (18.3%)**  **78 (15.0%)** | **19 (9.0%)**  **43 (8.2%)** | **2.00 [1.16;3.46]**  **1.78 [1.22;2.58]** | **0.013**  **0.003** |
